# A higher intramuscular fat in vastus medialis is associated with functional disabilities and symptoms in early stage of knee osteoarthritis: a case–control study

**DOI:** 10.1186/s13075-023-03048-0

**Published:** 2023-04-14

**Authors:** Masashi Taniguchi, Yoshihiro Fukumoto, Masahide Yagi, Tetsuya Hirono, Momoko Yamagata, Akihiro Asayama, Shogo Okada, Ryusuke Nakai, Masashi Kobayashi, Noriaki Ichihashi

**Affiliations:** 1 Human Health Sciences, Graduate School of Medicine, Kyoto University, 53-Kawahara-Cho, Shogoin, Sakyo-Ku, Kyoto, 606-8507 Japan; 2 Faculty of Rehabilitation, Kansai Medical University, Hirakata, Japan; 3 School of Health and Sport Science, Chukyo University, Aichi, Japan; 4 Research Fellow of Japan Society for the Promotion of Science, Tokyo, Japan; 5 Department of Rehabilitation, Japanese Red Cross Nagahama Hospital, Shiga, Japan; 6 Kyoto University Institute for the Future of Human Society, Kyoto, Japan; 7 Kobayashi Orthopaedic Clinic, Kyoto, Japan

**Keywords:** Early knee osteoarthritis, Intermuscular fat, Vastus medialis, Functional disabilities, Symptoms

## Abstract

**Background:**

The characteristics of muscle degeneration in individual quadriceps in early knee osteoarthritis (OA) and the association of muscle quantity and quality on knee dysfunction remain unclear. This study aimed to clarify the characteristics of muscle degeneration in individual quadriceps muscles in early knee OA and elucidate the association of muscle volume and intramuscular adipose tissue (intraMAT) with knee dysfunction, including functional disabilities, symptoms, and joint morphology.

**Methods:**

Fifty participants were categorized into early knee OA and healthy control groups. 3.0 T magnetic resonance imaging (MRI) using T1-weighted and Dixon methods and 3D SPACE in the thigh muscle and knee joint regions was performed. Quadriceps muscle volume, intraMAT, and whole-organ MRI score (WORMS) were assessed. The Knee Society Score (KSS) was used to evaluate functional disabilities and knee symptoms. Univariate analysis of variance was conducted with covariates to clarify the differences in muscle volume and intraMAT between the two groups. Multiple linear regression analyses were performed using the KSS function and symptom subcategories and WORMS as dependent variables and muscle volume, intraMAT, and the presence of early knee OA as independent variables, such as potential confounders.

**Results:**

The quadriceps intraMAT, especially in the vastus medialis (VM), was significantly higher in patients with early knee OA than in healthy controls. The VM intraMAT, not muscle volume, was significantly associated with KSS function [*B* =  − 3.47; 95% confidence interval [CI], − 5.24 to − 1.71; *p* < 0.001] and symptom scores [*B* =  − 0.63; 95% CI, − 1.09 to − 0.17; *p* = 0.008], but not with WORMS.

**Conclusion:**

These findings suggest that higher VM intraMAT is characteristic of quadriceps muscle degeneration in early knee OA and its increase is associated with functional disabilities and symptoms.

## Introduction

Knee osteoarthritis (OA) is one of the most common musculoskeletal disorders that worsen functional ability in older people. Early detection of knee OA pathology is important for preventing cartilage degeneration and worsening of symptoms. Since the definition of early-stage knee OA (early knee OA) was published in the 2010s [1, 2], clinical studies have used the classification criteria for early knee OA based on radiographic Kellgren-Lawrence (KL) grade 0–1 and clinical signs including poor function and pain. The development and progression of early knee OA by evaluating intra-articular lesions using magnetic resonance imaging (MRI) is an active area of research [3, 4]. A previous study [5] indicated that medial meniscal extrusion is a known risk factor for the progression of early knee OA, but the modifiable factors that could change with therapeutic intervention remain unclear. Recently, some studies [6–8] have suggested that intramuscular adipose tissue (intraMAT) infiltration into the quadriceps in patients with advanced knee OA is associated with muscle weakness and functional disabilities; thus, quantification of intraMAT has attracted attention for detecting knee OA pathology. As an increase in quadriceps intraMAT occurs earlier than loss of muscle quantity (i.e., muscle atrophy) in age-related changes [6, 9], assessment of intraMAT infiltration may also be useful in detecting early knee OA.

Chemical shift-based 2-point Dixon water-fat separation MRI techniques can be used for the quantification of intraMAT in localized regions [10, 11]. Dixon-MRI has high spatial resolution and is more advantageous for extracting intraMAT than T1-weighted methods, as measured by pixel intensity [12].

Only one study [6] has evaluated intraMAT in knee OA using the Dixon-MRI technique has been reported.

To the best of our knowledge, only one previous study [6], by Kumar et al., has evaluated intraMAT in knee OA using the Dixon-MRI technique. Their study [6] showed that intraMAT infiltration was significantly higher in the quadriceps of patients with knee OA than in healthy controls, whereas no significant difference was observed in other thigh muscles. Additionally, no difference between groups was observed in the anatomical cross-sectional area of all thigh muscles, implying that muscle atrophy in the quadriceps was not observed in knee OA [6]. Although these results are interesting, it is still unknown whether these changes occur in early knee OA and whether there are differences in these changes between the four individual quadriceps muscles.

Several previous studies [7, 13] have shown that a high intraMAT in the vastus medialis (VM) muscle is associated with the worsening of cartilage damage. Other previous studies [9, 14] evaluated intraMAT using B-mode ultrasound images and suggested that an increase in VM intraMAT was confirmed in knee OA patients, and higher fat content was associated with poor functional abilities and worsening symptoms. In summary, although the number of previous reports is limited, there is a close interaction between VM intraMAT and structural changes and functional disabilities in knee OA patients. Considering that an increase in intraMAT occurs earlier than muscle atrophy [9, 15], a higher VM intraMAT may characterize quadriceps muscle degeneration in early knee OA patients. However, to the best of our knowledge, no study has investigated both muscle volume and intraMAT of individual quadriceps muscles and clarified the relationship between muscle degeneration characteristics and knee dysfunction in early knee OA.

The present study aimed to determine (1) the characteristics of muscle degeneration in individual quadriceps in early knee OA and (2) the association of muscle volume and intraMAT on knee dysfunction, such as functional disabilities, symptoms, and joint morphology. We hypothesized that (1) the intraMAT in the VM muscle was increased in early knee OA patients compared to healthy controls, and (2) a higher VM intraMAT was associated with knee dysfunction.

## Patients and methods

### Study participants

A total of 52 older adults aged > 60 years (women, *n* = 23; mean age, 73.5 ± 6.6 years) residing in local communities in Kyoto and neighboring cities participated in this cross-sectional observational study. Before starting the study, the study procedures and aims were explained verbally, and all individuals provided written informed consent. The protocol was approved by the Ethics Committee of Kyoto University Graduate School and Faculty of Medicine (R1746).

The inclusion criteria were as follows: (1) no diagnosis of advanced knee OA (KL grade ≥ 2), (2) ability to walk without an assistive device, and (3) no general contraindications for MRI. The exclusion criteria were rheumatoid arthritis, knee osteonecrosis, neuromuscular disorders, history of lower-limb surgery, and cognitive impairment. Thereafter, according to the classification criteria, the participants were divided into early knee OA and healthy control groups. Early knee OA was defined as a KL grading scale of 1 or less, tenderness and/or stiffness around the knee, and symptomatic knee assessed using the Knee Society Score (KSS) symptom subcategory. The healthy control group included patients who did not meet the definition of early knee OA and those without knee symptoms.

### MRI acquisition

MRI scans of the right knee joint and thigh muscle were obtained using a 3.0 T MRI scanner (MAGNETOM Verio; Siemens AG, Germany). After resting for more than 15 min in a relaxed supine position, MRIs of the whole knee joint were acquired with a sequence corresponding to 3D SPACE with proton-density variable contrast using a body matrix coil and spine coil. The parameters were set as follows: slice thickness, 0.7 mm; repetition time (TR), 1000 ms; echo time (TE), 35 ms; field of view, 150 mm × 150 mm; voxel size, 0.59 mm × 0.59 mm × 0.7 mm. Additionally, MRIs of the entire thigh were obtained from the pelvis to the right tibial tuberosity and divided into three parts with a body matrix coil and a spine coil. Multi-slice T1-weighted MRIs of the thigh muscles were performed with the following parameters: [16] slice thickness, 4 mm; TR, 2820 ms; TE, 16 ms; optimized field of view, 320 mm × 240 mm; flip angle, 129°; voxel size, 0.5 mm × 0.5 mm × 4.0 mm. Two-point Dixon images with a slice thickness of 3 mm were acquired using the following sequences [17]: TR, 4.33 ms; TE1, 1.31 ms; TE2, 2.54 ms; optimized field of view, 286.4 mm × 365 mm; flip angle, 9°. Based on the water/fat chemical shift difference and consequently on their phase difference in signal intensity, water and fat images were produced using the two-point Dixon sequence. IntraMAT was calculated based on the signal intensities using the following equation: IntraMAT (%) = mean signal intensity of fat × 100/(mean signal intensity of fat + mean signal intensity of water) [17].

### Quantification of muscle volume and intraMAT

The region of interest (ROI) in individual quadriceps muscles was registered in T1-weighted and two-point Dixon images using Osirix MD (version 11.0; OsiriX, Geneva, Switzerland). To measure the muscle volume, the ROIs in the rectus femoris (RF), vastus intermedius (VI), vastus lateralis (VL), and VM were traced on each slice throughout the entire muscle length and measured as the muscle cross-sectional area (CSA, cm^2^). Then, the volume (cm^3^) of each slice was obtained by multiplying the muscle CSA by the slice thickness of 4 mm, and the muscle volume was calculated by summing the volume of each slice throughout the entire muscle length. The muscle volumes of the four individual quadriceps and total quadriceps were determined. IntraMAT was measured using two-point Dixon imaging. ROIs were carefully traced within the fascial borders of each muscle using a well-trained analyzer. The ROIs for RF, VI, and VL were constructed at the mid-thigh between the anterior superior iliac spine and the proximal end of the patella, and those for VM were at the distal 30% between the greater trochanter and the lateral femoral tuberosity. The intraMAT (%) was calculated from every 10 consecutive images with the measurement point mentioned above as the center, and the average value was obtained for the four individual quadriceps. The mean intraMAT of the quadriceps was determined as the average value of the four individual muscles.

### Assessment of the Kellgren-Lawrence grading scale and knee OA joint morphology

After obtaining 3D SPACE MRIs of the right knee, one orthopedic surgeon judged the OA severity of the tibiofemoral joint and then confirmed the criteria for radiographic early knee OA. The KL grade based on knee MRIs was evaluated using a grading system with the Noyes classification, adding the status of osteophytes, bone marrow edema, and subchondral cyst, and it was strongly correlated with that of X-ray evaluation [18].

Knee joint morphology was assessed using the whole-organ magnetic resonance imaging score (WORMS), a semi-quantified scoring method based on knee imaging [19]. Five features of the articular surface, such as cartilage, subarticular bone marrow, bone cyst, subarticular bone attrition, and osteophyte, were scored by a well-trained examiner. Fifteen regions of the patella, femur, and tibia were assessed using the original WORMS. The total WORMS was obtained by adding the scores for each feature, and a higher WORMS indicated greater severity of morphological degeneration.

### Self-reported knee function and symptom

Functional disabilities and symptoms were measured using the KSS 2011 Japanese edition, a self-administered assessment tool. The KSS comprises functional activities, symptoms, expectations, and satisfaction. This battery has demonstrated validity in the Japanese population, and two subcategories of functional activities and symptoms were used in the present study [20]. The KSS function score evaluates the activity status during daily living, such as standing, walking, and standard/advanced/discretionary activities. The maximum score on the KSS function was 100 points, and a higher score reflected better functional activities. Additionally, KSS symptoms, with a maximum score of 25 points, quantify knee stiffness and the degree of pain during walking and climbing stairs. Lower KSS symptoms indicate worsening knee pain, and a score of less than 23 is defined as a symptomatic knee [21].

### Clinical features of physical function and amount of physical activity

Gait speed (m/s) during the participant’s comfortable walking measured the time it took to pass the gait way at a distance of 10 m and was converted to speed by dividing by the time taken. The chair stand-30 test measured the number of repetitions with sit-to-stand for 30 s as much as possible. The knee extension strength test was performed twice using an isometric dynamometer (Isoforce GT-330; OG GIKEN Co., Japan) with knee flexion of 60°, and the maximum value was obtained by converting into a ratio of torque to body weight (Nm/kg). The average number of steps per day for 14 days was measured as the amount of physical activity in daily life using a pedometer with a triaxial accelerometer (ES-500; YAMASA, Japan).

### Statistical analysis

Continuous variables are shown as the mean ± standard deviation (SD), and categorical variables are presented as counts and percentages (*n*, %). Outcome measures and covariates were compared between the early knee OA and healthy control groups using an unpaired *t*-test or a chi-square test. Univariate analysis of variance (ANOVA) was conducted to clarify the differences in muscle volume and intraMAT between the early knee OA and healthy control groups. The adjusted mean differences between the two groups for each parameter were estimated by adjusting for age, sex, and body mass index (BMI). To determine the cut-off value of muscle degeneration parameters in early knee OA, receiver operating characteristic (ROC) curve analysis with the Youden index was conducted on the significant variable confirmed by group difference analysis. We performed three multiple linear regression analyses with the KSS function, symptoms, and WORMS as dependent variables. The regression analyses included quadriceps muscle volume, intraMAT, and the presence of early knee OA as independent variables, with adjustment variables for age, sex, and BMI. For secondary analyses, we also conducted another multiple linear regression as the independent variable with VM muscle volume and intraMAT to test our hypothesis. All statistical tests were performed using the SPSS software (version 25.0 for Windows, IBM Japan Inc., Japan). Statistical significance was set at *p* < 0.05.

## Results

Besides two excluded participants with blurred MRIs (obvious artifacts in images) or missing clinical data, 50 participants were divided into the early knee OA (*n* = 19) and healthy control groups (*n* = 31). The demographic characteristics are shown in Table 1. KSS function and symptom scores in the early knee OA group were significantly lower than those in the healthy control group, while there was no significant difference in WORMS scores between groups. There were no significant differences in muscle volume and intraMAT between the two groups, except for the VM intraMAT, which was higher in early knee OA than in healthy controls. Physical function and amount of physical activity were also not significantly different between the two groups.

**Table 1:** Clinical characteristics of early knee OA and healthy control groups

	Total	Control	Early knee OA	*p*-value
	*n* = 50	*n* = 31	*n* = 19	
Age, years	73.5 ± 6.6	73.8 ± 6.3	72.9 ± 7.3	0.882
Sex [women], *n* (%)	23 (46.0%)	14 (45.2%)	9 (47.4%)	0.879
Body mass index, kg/m^2^	20.8 ± 2.4	20.9 ± 2.2	20.7 ± 2.7	0.859
KSS function, /100	91.6 ± 12.5	95.5 ± 6.6	85.2 ± 16.8	0.003
KSS symptom, /25	22.7 ± 3.5	24.5 ± 0.9	19.8 ± 4.2	< 0.001
Total of WORMS	18.6 ± 13.1	17.8 ± 13.2	20.0 ± 13.3	0.563
OA severity; KL grade = 0, *n* (%)	19 (38.0%)	12 (38.7%)	7 (36.8%)	0.895
Muscle volume, cm^3^				
Quadriceps femoris	1108.4 ± 277.0	1093.2 ± 259.7	1133.2 ± 308.8	0.624
Rectus femoris	154.0 ± 40.8	149.4 ± 40.6	161.7 ± 41.1	0.305
Vastus intermedius	317.7 ± 85.6	320.3 ± 82.2	313.6 ± 93.0	0.792
Vastus lateralis	359.4 ± 92.8	348.6 ± 80.3	377.0 ± 110.4	0.298
Vastus medialis	277.2 ± 71.4	274.9 ± 69.9	281.0 ± 75.6	0.775
Intramuscular fat, %				
Quadriceps femoris	7.1 ± 1.8	6.7 ± 1.2	7.7 ± 2.3	0.073
Rectus femoris	5.7 ± 1.4	5.5 ± 1.1	6.1 ± 1.8	0.196
Vastus intermedius	7.6 ± 1.9	7.4 ± 1.4	7.9 ± 2.5	0.354
Vastus lateralis	8.2 ± 2.2	8.0 ± 1.8	8.5 ± 2.8	0.409
Vastus medialis	6.8 ± 2.2	6.0 ± 1.2	8.2 ± 2.8	< 0.001
Gait speed, m/s	1.6 ± 0.2	1.6 ± 0.2	1.5 ± 0.2	0.093
Chair stand-30, repetitions	27.2 ± 6.4	26.3 ± 7.1	28.6 ± 4.8	0.214
Knee extension strength, Nm/kg	2.3 ± 0.6	2.3 ± 0.6	2.4 ± 0.6	0.703
Steps (average 1000 steps per day)	8.3 ± 3.7	8.2 ± 3.4	8.4 ± 4.3	0.796

*KSS* Knee Scoring System, *WORMS* Whole-organ magnetic resonance imaging score, *KL* Kellgren-Lawrence

### The characteristics of muscle degeneration in the early knee OA group

Based on the results of univariate ANOVA with adjustment for covariates (Table 2), we observed that quadriceps intraMAT, not muscle volume, was significantly higher in the early knee OA group than in the healthy control (adjusted mean difference, 1.0%; 95% confidence interval [CI], 0.1 to 1.9%; *p* = 0.035). Further, for individual quadriceps muscles, the VM intraMAT in the early knee OA group was significantly higher than that in the healthy control (adjusted mean difference, 2.2%; 95% CI, 1.2 to 3.3%; *p* < 0.001), while intraMAT of the RF, VI, and VL did not confirm group differences. The volume of all four muscles also showed no significant differences between the two groups. ROC curve analysis indicated that the optimal cut-off point of the VM intraMAT for detecting early knee OA was 7.2%. This model on VM intraMAT had moderate prediction accuracy, with an area under the curve of 0.80, sensitivity of 0.63, and specificity of 0.81.

**Table 2:** Muscle volume and intraMAT differences between the early knee OA and healthy control groups

	Adjusted mean (SE)		*p*-value	Adjusted mean difference [95% CI]
	Healthy control	Early knee OA		Early knee OA against control
Muscle volume, cm^3^				
Quadriceps femoris	1089.6 ± 25.4	1139.2 ± 32.5	0.235	49.6 [− 33.5, 132.7]
Rectus femoris	149.1 ± 4.5	162.1 ± 5.7	0.080	13.1 [− 1.6, 27.7]
Vastus intermedius	319.2 ± 8.2	315.4 ± 10.4	0.777	− 3.8 [− 30.5, 23.0]
Vastus lateralis	347.3 ± 10.1	379.2 ± 12.9	0.058	31.8 [− 1.2, 64.8]
Vastus medialis	274.0 ± 7.0	282.5 ± 9.0	0.460	8.5 [− 14.5, 31.6]
IntraMAT, %				
Quadriceps femoris	6.7 ± 0.3	7.7 ± 0.4	0.035	1.0 [0.1, 1.9]
Rectus femoris	5.5 ± 0.2	6.1 ± 0.3	0.138	0.6 [− 0.2, 1.3]
Vastus intermedius	7.4 ± 0.3	7.9 ± 0.4	0.287	0.5 [− 0.5, 1.6]
Vastus lateralis	7.9 ± 0.4	8.6 ± 0.5	0.253	0.7 [− 0.5, 1.8]
Vastus medialis	6.0 ± 0.3	8.2 ± 0.4	< 0.001	2.2 [1.2, 3.3]

*OA* Osteoarthritis, *SE* Standard error, *95% CI* 95% confidence interval, *IntraMAT* Intramuscular fat

The adjusted mean differences were calculated with adjustments for age, sex, and BMI and presented as the early knee OA group values against the healthy control group

### Association of muscle volume and intraMAT on KSS function, symptom, and WORMS

Associations of quadriceps muscle volume, intraMAT, and the presence of early knee OA with KSS function, symptoms, and WORMS are presented in Table 3. In the multiple regression analysis, quadriceps intraMAT and the presence of early knee OA were selected as significant variables for the KSS function score. A 1% increase in quadriceps intraMAT was significantly associated with a 3.88-point reduction in the KSS function score (95% CI, 1.82 to 5.93 points, *p* < 0.001) after adjusting for covariates, but the muscle volume was not. In contrast, the presence of early knee OA, but not quadriceps muscle volume or intraMAT, was associated with the KSS symptom score. There were no significant variables associated with WORMS.

**Table 3:** Association of quadriceps muscle volume and intraMAT with KSS function, symptom, and WORMS

	*B*	95% CI	*β*	*p*-value
Association with KSS function scores				
Quadriceps muscle volume	− 0.00	[− 0.02, 0.02]	− 0.03	0.913
Quadriceps intraMAT	− 3.88	[− 5.93, − 1.82]	− 0.54	< 0.001
Presence of early knee OA	− 6.79	[− 13.21, − 0.38]	− 0.30	0.038
Age	− 0.02	[− 0.49, 0.44]	− 0.01	0.917
Sex	− 0.77	[− 10.99, 9.46]	− 0.03	0.881
Body mass index	− 0.03	[− 1.82, 1.75]	− 0.01	0.970
Association with KSS symptom scores				
Quadriceps muscle volume	0.00	[− 0.01, 0.01]	0.07	0.776
Quadriceps intraMAT	− 0.52	[− 1.07, 0.03]	− 0.26	0.061
Presence of early knee OA	− 4.28	[− 5.92, − 2.57]	− 0.60	< 0.001
Age	0.05	[− 0.07, 0.17]	0.09	0.415
Sex	0.40	[− 2.32, 3.13]	0.06	0.768
Body mass index	− 0.03	[− 0.51, 0.45]	− 0.02	0.899
Association with WORMS				
Quadriceps muscle volume	− 0.01	[− 0.04, 0.02]	− 0.20	0.517
Quadriceps intraMAT	− 1.19	[− 3.80, 1.43]	− 0.16	0.365
Presence of early knee OA	4.65	[− 3.52, 12.81]	0.17	0.258
Age	0.76	[0.17, 1.35]	0.38	0.013
Sex	− 2.42	[− 15.44, 10.60]	− 0.09	0.709
Body mass index	1.69	[− 0.59, 3.96]	0.31	0.142

Multiple linear regression analyses were conducted with KSS function, symptom, and WORMS as the dependent variable and quadriceps muscle volume, intraMAT, and the presence of early knee OA as the dependent variable, with adjustments for age, sex, and BMI

*KSS* Knee Scoring System, *WORMS* Whole-organ magnetic resonance imaging scoring, *OA* Osteoarthritis, *SE* Standard error, *95% CI* 95% confidence interval, *IntraMAT* Intramuscular fat

Table 4 presents the results of the secondary analyses which entered VM muscle volume, intraMAT, and the presence of early knee OA as the independent variables. VM intraMAT was selected as a significant variable for the KSS function score, whereas VM intraMAT and the presence of early knee OA were selected for the KSS symptom score. A higher VM intraMAT was significantly associated with KSS function (*B* =  − 3.47; 95% CI, − 5.24 to − 1.71; *p* < 0.001) and symptoms (*B* =  − 0.63; 95% CI, − 1.09 to − 0.17; *p* = 0.008). In the secondary analyses, there were no significant variables associated with WORMS.

**Table 4:** Association of VM muscle volume and intraMAT with KSS function, symptom, and WORMS

	*B*	95% CI	*β*	*p*-value
Association with KSS function score				
VM muscle volume	− 0.00	[− 0.09, 0.08]	− 0.01	0.955
VM intraMAT	− 3.47	[− 5.24, − 1.71]	− 0.62	< 0.001
Presence of early knee OA	− 2.91	[− 10.05, 4.22]	− 0.11	0.415
Age	− 0.05	[− 0.50, 0.39]	− 0.03	0.807
Sex	− 0.76	[− 10.21, 8.68]	− 0.03	0.871
Body mass index	− 0.11	[− 1.83, 1.61]	− 0.02	0.897
Association with KSS symptom score				
VM muscle volume	− 0.00	[− 0.02, 0.02]	0.01	0.900
VM intraMAT	− 0.63	[− 1.09, − 0.17]	− 0.40	0.008
Presence of early knee OA	− 3.33	[− 5.20, − 1.47]	− 0.46	0.001
Age	0.05	[− 0.07, 0.17]	0.09	0.386
Sex	0.12	[− 2.35, 2.59]	0.02	0.923
Body mass index	0.06	[− 0.39, 0.51]	0.04	0.777
Association with total WORMS				
VM muscle volume	− 0.06	[− 0.16, 0.05]	− 0.30	0.295
VM intraMAT	− 1.67	[− 3.93, 0.59]	− 0.28	0.143
Presence of early knee OA	7.21	[− 1.93, 16.36]	0.27	0.119
Age	0.77	[0.20, 1.34]	0.39	0.009
Sex	− 3.51	[− 15.61, 8.60]	− 0.14	0.562
Body mass index	2.06	[− 0.15, 4.26]	0.37	0.066

Multiple linear regression analyses were conducted with KSS function, symptom, and WORMS as the dependent variable and VM muscle volume, intraMAT, and the presence of early knee OA as the dependent variable, with adjustments for age, sex, and BMI

*KSS* Knee Scoring System, *WORMS* Whole-organ magnetic resonance imaging scoring, *VM* Vastus medialis, *OA* Osteoarthritis, *SE* Standard error, *95% CI* 95% confidence interval, *IntraMAT* Intramuscular fat

## Discussion

To the best of our knowledge, this study is the first to clarify the characteristics of muscle degeneration in early knee OA. In agreement with our hypothesis, quadriceps intraMAT, especially in the VM, was significantly higher in the early knee OA group than in healthy controls. Moreover, multiple regression analysis indicated that a higher quadriceps intraMAT was associated with worsening KSS function, partially supporting our hypothesis. Notably, this association was remarkably confirmed in VM intraMAT, and a higher VM intraMAT was associated with both functional disabilities and worse symptoms but not with joint morphology. These findings suggest that VM intraMAT is characteristic of quadriceps muscle degeneration in early knee OA and its increase is associated with functional disabilities and symptoms.

The current study found that the VM intraMAT increased even in early knee OA, and the ROC analysis showed a VM intraMAT of 7.2% for detecting early knee OA. Although the sensitivity was slightly low, specificity was high; thus, it was suggested that evaluation of the VM intraMAT among the four individual quadriceps muscles was useful for differentiating early knee OA. There is already evidence in advanced knee OA that intraMAT infiltration occurs prior to muscle atrophy in the quadriceps [6, 9, 22] and that the loss of muscle quality in the VM, such as higher intraMAT, characterizes quadriceps degeneration [8]. Some factors may contribute to the higher VM intraMAT. One factor is the mesenchymal progenitor cells, which are associated with ectopic adipogenesis of the VM in patients with advanced knee OA [23]. A previous study in mice showed that chronic inflammation within the muscles also promotes muscle degeneration [24]. The inflammatory cytokine IL-6 is amplified in patients with knee OA, and higher IL-6 levels are associated with worsened knee pain [25, 26]. Considering that patients with early knee OA have pain and slight knee degeneration, the aforementioned response may have arisen even in early knee OA. Additionally, the VM is a dynamic stabilizer of the knee and attaches closer to the painful site than to other quadriceps muscles [27, 28]. Therefore, biomechanical and anatomical factors and added biological factors are associated with a higher VM intraMAT. This mechanism is currently unknown; therefore, further studies are needed to elucidate why VM intraMAT increases in early knee OA.

An increase in VM intraMAT was associated with lower KSS function and symptoms, but not WORMS, in early knee OA. Although a higher VM intraMAT was reported to be one of the risk factors for cartilage damage in a longitudinal study [6], VM intraMAT was not related to WORMS in this study. This discrepancy could be due to differences in the severity of knee OA; previous studies [7, 13] included patients with advanced knee OA, but the current study targeted participants with early knee OA. The association of VM intraMAT with KSS function scores was consistent with a previous study in advanced knee OA [14]. Given that the minimum clinically significant difference in KSS function scores after total knee arthroplasty was 4.1 points [29], our findings that quadriceps 1% higher intraMAT was associated with a 3.88-point reduction in KSS functional score appear to be clinically significant. Notably, our findings suggested that VM intraMAT had a stronger relationship with KSS scores than quadriceps intraMAT. As the presence of early OA was not significant in the regression model using VM intraMAT, a higher VM intraMAT seemed to play a mediating role in the relationship between early knee OA and functional disabilities. Our results also showed that a higher VM intraMAT was associated with the worsening of KSS symptoms. Although it is known that an increase in intraMAT of the VM is associated with knee pain in advanced knee OA [14], the current study is the first to confirm similar associations in the pre-stage of knee OA development. As this study had a cross-sectional design, the causal relationship between knee symptoms and VM intraMAT infiltration in early knee OA could not be determined. As a clinical suggestion, it seems likely that VM intraMAT is meaningful for preventing functional disability and symptoms of knee osteoarthritis because, with muscle strength training, a higher intraMAT infiltration improves.

There are several limitations to this study. First, KL grade was converted based on the MRI grading system, such as the Noyes classification, to define early knee OA. Although the validity of this classification has been demonstrated, it may differ from the classification of early knee OA based on the anteroposterior weight-bearing X-ray view [18]. Second, the participants of this study were community-dwelling older adults. Thus, we assumed that our study participants were functionally better than individuals with early knee OA who visited an orthopedic clinic. No differences in WORMS or physical function were observed between the early knee OA and healthy control groups. Nonetheless, it is important to note that a higher intraMAT was identified in early knee OA.

## Conclusions

The intraMAT of the quadriceps, especially the VM, was significantly increased in patients with early knee OA compared with that in healthy controls. Furthermore, higher VM intraMAT was associated with poor functional abilities and symptoms. Therefore, these findings suggest that higher VM intraMAT is characteristic of quadriceps muscle degeneration in early knee OA and its increase is associated with functional disabilities and symptoms.

## Data Availability

The datasets generated during and/or analyzed during the current study are not publicly available due to ethical restrictions but are available from the corresponding author upon reasonable request.

## Abbreviations

ANOVA: Analysis of variance
BMI: Body mass index
CI: Confidence interval
CSA: Cross-sectional area
IntraMAT: Intramuscular adipose tissue
KL: Kellgren-Lawrence
KSS: Knee Scoring System
MRI: Magnetic resonance imaging
OA: Osteoarthritis
RF: Rectus femoris
ROC: Receiver operating characteristic
ROI: Region of interest
SD: Standard deviation
TE: Echo time
TR: Repetition time
VI: Vastus intermedius
VL: Vastus lateralis
VM: Vastus medialis
WORMS: Whole-organ magnetic resonance imaging score
